# Tocilizumab for severe and refractory mucous membrane pemphigoid

**DOI:** 10.1111/jdv.70510

**Published:** 2026-05-27

**Authors:** Billal Tedbirt, Maud Maho‐Vaillant, Julie Gueudry, Marie‐Laure Golinski, Marion Marcaillou, Blanche Bergeret, Guillaume Chambon, Pierre Bouchetemble, Soumeya Bekri, Abdellah Tebani, Hervé Maillard, Cécile Morice, Anais Vautier, Edgar‐Pierre Libert, Christelle Le Roux‐Villet, Marion Carrette, Olivier Boyer, Vivien Hébert, Sébastien Calbo, André Gillibert, Pascal Joly

**Affiliations:** ^1^ Department of Dermatology, French Reference Center for Auto Immune Blistering Diseases Rouen University Hospital, Normandie University Rouen France; ^2^ Univ Rouen Normandie, Inserm, Normandie Univ, PANTHER UMR 1234 Rouen France; ^3^ Department of Ophthalmology Rouen University Hospital Rouen France; ^4^ Department of Dermatology Toulouse University Hospital Toulouse France; ^5^ Department of Dermatology CHU Nîmes Nîmes France; ^6^ Department of Otolaryngology‐Head and Neck Surgery CHU Nîmes Nîmes France; ^7^ Department of Otolaryngology‐Head and Neck Surgery CHU Rouen Rouen France; ^8^ Department of Metabolic Biochemistry Normandie University, UNIROUEN, INSERM U1245, CHU Rouen Rouen France; ^9^ Department of Dermatology Hôpital du Mans Le Mans France; ^10^ Department of Dermatology CHU Caen Caen France; ^11^ Department of Ophthalmology Angers University Hospital Angers France; ^12^ Department of Dermatology Angers University Hospital Angers France; ^13^ Department of Dermatology & Reference Center for Autoimmune Bullous Diseases Avicenne University Hospital, AP‐HP, Sorbonne‐Paris‐Nord University Bobigny France; ^14^ Department of Immunology and Biotherapy Rouen University Hospital Rouen France; ^15^ Department of Biostatistics CHU Rouen Rouen France

**Keywords:** benign mucous membrane, bullous pemphigoid, mucous membrane pemphigoid, pemphigoid, proteomics, tocilizumab

## Abstract

**Background:**

Mucous membrane pemphigoid (MMP) is a rare autoimmune blistering disease involving mucous membranes with a potentially devastating fibrosing course. Laryngotracheal and/or oesophageal involvement may lead to life‐threatening complications, while corneal involvement may lead to blindness.

**Objectives:**

To characterize the serum cytokine profile of MMP and to evaluate the efficacy of tocilizumab in nine cases of severe MMP according to the serum levels of IL‐6.

**Methods:**

Sera from MMP patients were compared to sex‐ and age‐matched healthy donors (HD) and patients with bullous pemphigoid (BP), using two proteomic assays (Olink and Luminex). The Olink assay included 40 MMP, 40 BP and 40 HD sera, and the Luminex assay included 63 MMP, 31 BP and 24 HD sera. Based on these findings, nine patients with severe and refractory MMP received tocilizumab on a compassionate use basis after failure of standard therapies.

**Results:**

Proteomic analyses revealed high serum levels of IL‐6, IL‐8, MIP‐1α/CCL3 and MIP‐1β/CCL4 in MMP compared to BP and HD. Patients with MMP who achieved complete remission during follow‐up had lower baseline serum levels of IL‐6 (*p* < 0.0001), IL‐8 (*p* = 0.0382), MIP‐1α/CCL3 (*p* = 0.0005) and MIP‐1β/CCL4 (*p* = 0.0009) than those with persistent disease activity.

Nine patients with severe and refractory MMP were treated with tocilizumab. Their mean MMPDAI score decreased from 14.8 ± 6.6 to 4.2 ± 3.2 (*p* = 0.009). At the last evaluation, seven patients (78%) were in partial remission, one (11%) in complete remission and one (11%) recently treated achieved control of disease activity. Baseline IL‐6 was elevated in five of eight tested patients; all three with normal IL‐6 levels improved clinically. Four non‐severe adverse events were observed (two neutropenias, one thrombocytopenia, one hepatic cytolysis).

**Conclusions:**

Proteomic analyses identified a distinct MMP cytokine profile with increased IL‐6, IL‐8, MIP‐1α/CCL3 and MIP‐1β/CCL4, supporting IL‐6 targeting, with tocilizumab as a potential option in severe refractory MMP.

## Plain Language Summary

**What Is the Disease?:**

Mucous membrane pemphigoid (MMP) is a rare autoimmune blistering disease involving mucous membranes with a potentially devastating fibrosing course. Laryngotracheal involvement may lead to life‐threatening complications, while corneal involvement may lead to blindness.

**Location of the Study:**

This study was conducted in France.

**Aim of the Study:**

To characterize the serum cytokine profile of MMP and to evaluate the efficacy of tocilizumab in nine cases of severe MMP.

**Methodology:**

Sera from patients with MMP were compared to sex‐ and age‐matched healthy donors and patients with bullous pemphigoid using two proteomic assays (Olink: 40 MMP, 40 BP, 40 healthy donors; Luminex: 63 MMP, 31 BP, 24 healthy donors). Based on these findings, nine patients with severe and refractory MMP received tocilizumab on a compassionate use basis after failure of standard therapies.

**Key Findings:**

Proteomic analyses revealed high serum levels of IL‐6, IL‐8, MIP‐1α/CCL3 and MIP‐1β/CCL4 in MMP compared to bullous pemphigoid and healthy donors. Patients who achieved complete remission had lower baseline levels of these cytokines than those with persistent disease activity. After tocilizumab treatment, all nine patients showed clinical improvement with a significant reduction in disease severity. No severe adverse event was observed.

**Conclusions and Practical Implications:**

Tocilizumab may be considered after the failure of validated treatments in patients with severe MMP.

## Graphical abstract

Elevated IL‐6 levels identified in mucous membrane pemphigoid support IL‐6 blockade, with tocilizumab demonstrating clinical benefit in nine patients with severe and refractory mucous membrane pemphigoid.

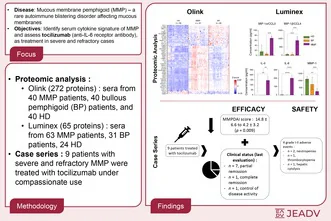

**Why was the study undertaken?**

Does the serum of patients with mucous membrane pemphigoid (MMP) have a distinct cytokine profile, and does this profile indicate a specific cytokine that could serve as a therapeutic target?

**What does this study add?**

Using proteomic analyses, we identified a specific cytokine signature in MMP relative to patients with bullous pemphigoid and healthy donors, including increased serum levels of IL‐6, IL‐8, MIP‐1α/CCL3 and MIP‐1β/CCL4.

Based on these findings, we successfully treated nine patients with severe and refractory MMP with tocilizumab, an anti‐IL‐6 receptor monoclonal antibody, without any severe adverse events.

**What are the implications of this study for disease understanding and/or clinical care?**

Tocilizumab may be considered after the failure of validated treatments in patients with severe MMP, particularly in those at risk of treatment‐related complications or contraindications to conventional immunosuppressants.

## INTRODUCTION

Mucous membrane pemphigoid (MMP) is a rare autoimmune blistering disease of the dermal‐epidermal junction, characterized by predominant or exclusive mucosal involvement.^1^, ^2^ The disease affects the oral, nasopharyngeal, laryngotracheal, genital, oesophageal, anal and ocular mucous membranes. Epithelial detachment from the laryngotracheal area may lead to life‐threatening suffocation crises. Fibrosing lesions may also result in life‐threatening laryngeal, tracheal and oesophageal stenosis.^1^ Ocular complications include severe cicatrizing conjunctivitis, ultimately leading to reduced vision due to corneal epithelial defects, neovascularization or even perforation. Severe forms of MMP are usually treated with oral or intravenous corticosteroids, cyclophosphamide or, more recently, rituximab, while less severe forms are treated with dapsone or mycophenolate mofetil.^1^, ^2^ Unfortunately, these treatments do not always prevent the occurrence of severe fibrosing sequelae. Additionally, both cyclophosphamide and rituximab may be poorly tolerated in elderly patients, with a high risk of severe infection. Intravenous immunoglobulins have also been proposed for severe forms, although evidence supporting their efficacy is limited.^1^, ^2^

The pathogenesis of MMP remains poorly understood.^3^ Serum antibodies are directed against several proteins of the dermal‐epidermal junction, including BP180, laminin 332, integrin α6β4 and type VII collagen.^1^, ^2^, ^3^ Further pathological processes involve polymorphonuclear neutrophils and fibroblasts.^4^, ^5^, ^6^ The cytokine profile in MMP has been poorly studied, with a few studies on ocular forms showing increased levels of profibrotic cytokines in ocular surface samples.^4^, ^7^, ^8^, ^9^ In the present study, we performed extensive proteomic serum analyses to identify a cytokine profile, which might be characteristic of MMP as compared with healthy donors and patients with bullous pemphigoid. As we observed that many patients with MMP had increased interleukin (IL)‐6 serum levels, we treated nine patients with severe and refractory MMP with tocilizumab, an anti‐IL‐6 receptor monoclonal antibody, under compassionate use after failure of standard therapies.

## METHODS

### Proteomic analysis

Sera from 40 patients with MMP, included in the RITUX‐MMP clinical trial (NCT03295383), were analysed and compared with samples from 40 sex‐ and age‐matched healthy donors obtained from Biovit (West Sussex, UK), and 40 newly diagnosed patients with bullous pemphigoid (local ‘autoimmune blistering disease’ collection: DC‐2021‐4499). Importantly, all patients with MMP included in the proteomic analysis had active disease at the time of serum collection and were rituximab‐naïve at the time of sampling. All sera were stored at −80°C and had never been defrosted prior to analysis. Written informed consent was obtained from all subjects participating in the study. Proteomic analyses were performed using the Olink Proteomics Target 96 Immune Response, Target 96 Inflammation and Target 96 Metabolism panels (Olink Proteomics, Uppsala, Sweden) (www.olink.com) on the Olink Signature Q100 system. The final dataset consisted of 272 unique proteins which were analysed across 120 serum samples.

Sera from 63 patients with MMP (including 40 sera also analysed by proteomic), 24 healthy donors (0 in common) and 31 patients with bullous pemphigoid (16 in common) were analysed by Luminex technology to measure 65 markers including various cytokines, chemokines, growth factors and soluble receptors, using ProcartaPlex Human Immune Monitoring 65‐plex Panel (Thermo Fisher Scientific) according to the manufacturer's instructions (www.thermofisher.com). The panel measured a total of 65 markers, including cytokines (i.e., G‐CSF, GM‐CSF, IFN alpha, IFN gamma, IL‐1 alpha, IL‐1 beta, IL‐2, IL‐3, IL‐4, IL‐5, IL‐6, IL‐7, IL‐8 (CXCL8), IL‐9, IL‐10, IL‐12p70, IL‐13, IL‐15, IL‐16, IL‐17A (CTLA‐8), IL‐18, IL‐20, IL‐21, IL‐22, IL‐23, IL‐27, IL‐31, LIF, M‐CSF, MIF, TNFα, TNFβ and TSLP), chemokines (i.e., BLC (CXCL13), ENA‐78 (CXCL5), Eotaxin (CCL11), Eotaxin‐2 (CCL24), Eotaxin‐3 (CCL26), Fractalkine (CX3CL1), Gro‐alpha (CXCL1), IP‐10 (CXCL10), I‐TAC (CXCL11), MCP‐1 (CCL2), MCP‐2 (CCL8), MCP‐3 (CCL7), MDC (CCL22), MIG (CXCL9), MIP‐1α (CCL3), MIP‐1β (CCL4), MIP‐3α (CCL20) and SDF‐1α (CXCL12)), growth factors and regulators (i.e., FGF‐2, HGF, MMP‐1, NGF beta, SCF and (VEGF‐A) and soluble receptors, i.e., APRIL, BAFF, CD30, CD40L (CD154), IL‐2R (CD25), TNF‐RII, TRAIL (CD253) and TWEAK). Healthy donor sera were analysed in the present study using the same Olink and Luminex protocols as patient samples, and were included across assay runs.

### Case series

We retrospectively extracted data from nine patients with severe and refractory MMP treated with tocilizumab after failure of standard therapies. The diagnosis of MMP was confirmed according to the European guidelines.^1^, ^2^ Extracted data included demographics, prior treatments, tocilizumab regimen, concomitant treatments, clinical response, disease severity (assessed using the Mucous Membrane Pemphigoid Disease Area Index [MMPDAI] score^10^) and adverse events (assessed according to the Common Terminology Criteria for Adverse Events [CTCAE]). Clinical response was assessed in accordance with the consensus definitions^10^: control of disease activity (CDA) (when new inflammatory lesions stop appearing and established ones begin to heal), partial remission (PR) (transient new lesions healing within 1 week), complete remission (CR) (no new or persistent lesions for at least 2 months) and relapse (appearance of 3 or more new lesions per month that fail to heal within 1 week, or progression of existing lesions after disease control). Serological tests included BP180 and BP230 ELISA (Euroimmun Lübeck, Germany), IgG indirect immunofluorescence on salt‐split skin and BIOCHIP testing for anti‐laminin 332, anti‐laminin β4 and anti‐collagen VII autoantibodies (Euroimmun Lübeck, Germany), which were performed in all sera. Serum IL‐6 level was measured shortly before initiation of tocilizumab in eight of nine patients. Follow‐up IL‐6 measurement was performed during treatment in two patients. Local ethics committee approval was obtained for retrospective data collection and analysis.

### Statistical analysis

For the proteomic analysis, two comparisons were performed: MMP versus healthy donors, and MMP versus bullous pemphigoid using two Benjamini‐Yekutieli procedures with false discovery rates (FDR) at 5% and 272 × 2 Mann–Whitney tests. An over‐representation analysis (ORA) of pathways was performed by one‐sided Fisher's exact tests. From the literature of molecular biology studies, large databases of genes related to the same function or sharing molecular structure have been built, such as the KEGG database. These databases define pathways as named lists of genes. An ORA identifies pathways that contain a higher number of overexpressed or underexpressed genes. For the pathway analysis, 3 databases of gene sets were used; all of the 29 gene sets containing at least 10 genes of our panels were used. These three databases of gene sets included gene ontology biological process (GOBP), Hallmark gene sets and KEGG gene sets. An over‐representation analysis (ORA) was performed by one‐sided Fisher's exact tests, looking for a higher percentage of differentially expressed genes in genes of the gene set than in genes outside of the gene set, with correction by a Benjamini‐Yekutieli procedure.

For the Luminex analysis, we used Mann–Whitney tests with FDR control at 20% by the Benjamini‐Yekutieli procedure. Three markers (IL‐23, IL‐31, TNF‐β) with no variance in HD were excluded. The remaining 62 markers were compared between patients with MMP and HD using 62 Mann–Whitney tests with FDR control at 20% by the Benjamini‐Yekutieli procedure. Similarly, MMP and bullous pemphigoid were compared with a second FDR at 20%; the area under the receiver operating characteristic (ROC) curves was computed without overfitting correction.

## RESULTS

### Proteomic analyses

**FIGURE 1:**
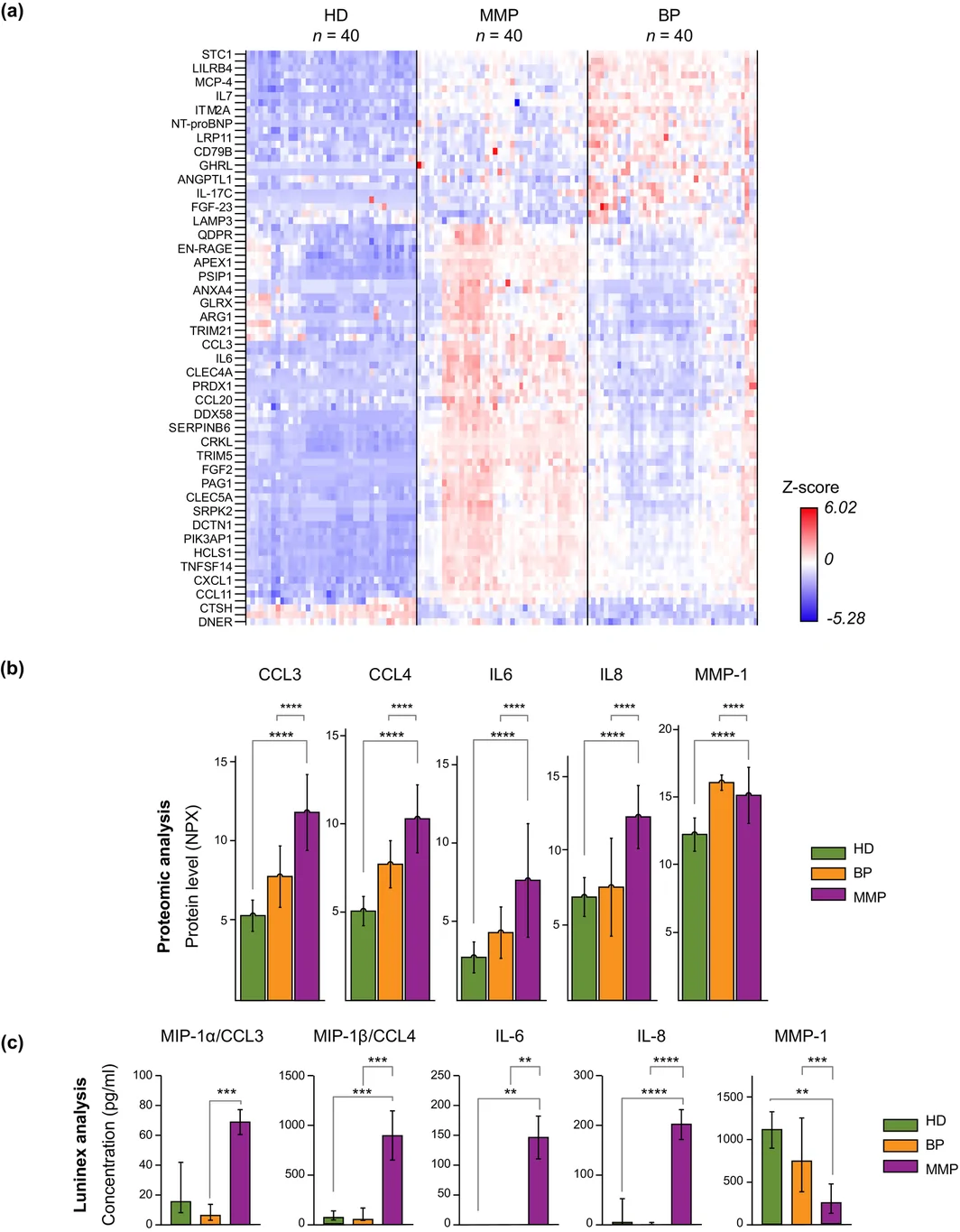
Differential protein expression analysis. (a) Heatmap showing the most significant proteins (FDR 1% for MMP versus BP) for each individual (*n* = 40 individuals for each subgroup, that is, HD, MMP and BP subgroups). (b) Proteomic (NPX ± standard error of the mean [SEM]) and Luminex (mean ± SEM) results showing the expression levels of five proteins (i.e., MIP‐1α/CCL3, MIP‐1β/CCL4, IL‐6, IL‐8 and MMP‐1). *p < 0.05; **p < 0.01; ***p < 0.001; ****p < 0.0001. *p*‐values were Benjamini‐Yekutieli adjusted. BP, bullous pemphigoid; HD, healthy donors; MMP, mucous membrane pemphigoid; NPX, normalized protein expression.

Among the 272 proteins analysed for proteomic expression, 179 and 66 were significantly increased (FDR 5%) in the serum of patients with MMP compared to healthy donors and patients with bullous pemphigoid, respectively. Conversely, the serum levels of 13 and 39 proteins were decreased in patients with MMP relative to healthy donors and patients with bullous pemphigoid, respectively (Figure S1 and Tables S1, S2). A heatmap of the most significant proteins (FDR 1% for MMP versus bullous pemphigoid) for each individual is shown in Figure 1. Pathway analysis revealed a significant association of the chemokine signalling and the nucleotide‐binding and oligomerization (NOD)‐like receptor signalling pathways in patients with MMP compared to healthy donors (adjusted *p* < 0.0001) (Table S3).

Using a Luminex assay, we then performed the quantitative measurement of 65 markers related to immune response, to compare with the proteomic analysis results. Comparison of MMP with healthy donors showed significantly higher levels of IL‐8 (area under curve [AUC] = 0.84, FDR adjusted *p* < 0.0001), macrophage inflammatory protein (MIP)‐1β/CCL4 (AUC = 0.80, adjusted *p* = 0.0005), IL‐6 (AUC = 0.77, adjusted *p* = 0.001) and IL‐16 (AUC = 0.76, adjusted *p* = 0.004) (Table S4 and Figure S2). The same analysis comparing MMP and bullous pemphigoid showed higher levels of IL‐8 (AUC = 0.90, adjusted *p* < 0.0001), MIP‐1β/CCL4 (AUC = 0.80, adjusted *p* = 0.0003), MIP‐1α/CCL3 (AUC = 0.78, adjusted *p* = 0.0005), IL‐1a (AUC = 0.77, adjusted *p* = 0.0006), IL‐6 (AUC = 0.74, adjusted *p* = 0.002) and IL‐9 (AUC = 0.68, adjusted *p* = 0.049) and lower levels of IL2‐R (AUC = 0.17, adjusted *p* < 0.0001), MMP‐1 (AUC = 0.21, adjusted *p* = 0.0004), APRIL (AUC = 0.29, adjusted *p* = 0.039) and CD30 (AUC = 0.27, adjusted *p* = 0.01) (Table S5 and Figure S3). The increase of IL‐8, MIP‐1β/CCL4, IL‐6 and MIP‐1α/CCL3 and the decrease of MMP‐1 in MMP versus bullous pemphigoid sera using Luminex were consistent with the results of proteomic analysis: IL‐8 (AUC = 0.887, adjusted *p* < 0.0001), MIP‐1β/CCL4 (AUC = 0.868, adjusted *p* < 0.0001), IL‐6 (AUC = 0.813, adjusted *p* < 0.0001), MIP‐1α/CCL3 (AUC = 0.909, adjusted *p* < 0.0001) and MMP‐1 (AUC = 0.221, adjusted *p* = 0.0003) (Table S2). By Olink proteomics, the most significantly elevated proteins in MMP relative to BP included CCL3/MIP‐1α (AUC = 0.909, adjusted *p* < 0.001), IL‐8 (AUC = 0.887, adjusted *p* < 0.001), CCL4/MIP‐1β (AUC = 0.868, adjusted *p* < 0.001) and IL‐6 (AUC = 0.813, adjusted *p* < 0.001), while the most elevated in BP relative to MMP included LILRB4 (AUC = 0.110, adjusted *p* < 0.001), CCL23 (AUC = 0.144, adjusted *p* < 0.001), CX3CL1 (AUC = 0.151, adjusted *p* < 0.001) and MCP‐4/CCL13 (AUC = 0.209, adjusted *p* < 0.001) (Table S2).

The characteristics of patients included in the proteomic analyses are presented in Table 1. At baseline, serum levels of IL‐6, IL‐8, MIP‐1α/CCL3 and MIP‐1β/CCL4 were identified as the only cytokines significantly elevated in MMP versus BP across both Olink and Luminex analyses (adjusted *p* < 0.05, AUC >0.5). Additionally, their levels did not differ significantly between treatment‐naïve (*n* = 29) and previously treated (*n* = 34) patients with MMP (Figure S4). However, patients who achieved CR (*n* = 24) after treatment had significantly lower levels of IL‐6 (*p* < 0.0001), IL‐8 (*p* = 0.0382), MIP‐1α/CCL3 (*p* = 0.0005) and MIP‐1β/CCL4 (*p* = 0.0009) than patients with persistent active disease (*n* = 20) (Figure S5).

**TABLE 1:** Characteristics of patients included in the proteomic analyses.

Characteristic	Mucous membrane pemphigoid group		Bullous pemphigoid group	
	Olink analyses (*n* = 40)*	Luminex analyses (*n* = 63)*	Olink analyses (*n* = 40)**	Luminex analyses (*n* = 31)**
Age, mean (SD), y	68.9 (11.2)	67.5 (10.2)	80.6 (12.5)	78.1 (11.7)
Sex, *n* (%)				
Female	19 (48%)	33 (52%)	26 (65%)	16 (52%)
Male	21 (52%)	30 (48%)	14 (35%)	15 (48%)
Severity score (MMPDAI or BPDAI scores), mean (SD)	22.6 (21.6)	23.3 (20.8)	41.5 (29.5)	59.8 (42.1)
Mucosal involvement, *n* (%)				
Oral mucosa	37 (93%)	57 (90%)	8 (20%)	4 (13%)
Ocular mucosa	12 (30%)	24 (38%)	0	0
Pharyngeal mucosa	11 (28%)	21 (33%)	0	0
Laryngeal mucosa	18 (45%)	27 (43%)	0	0
Comorbidities, *n* (%)				
Hypertension	13 (33%)	19 (30%)	26 (65%)	23 (74%)
Diabetes	7 (18%)	10 (16%)	12 (30%)	12 (39%)
Stroke	2 (5%)	2 (3%)	5 (13%)	2 (6%)
Dyslipidaemia	3 (8%)	3 (5%)	12 (30%)	14 (45%)
Cancer/haematologic malignancy	1 (3%)	1 (2%)	5 (13%)	3 (10%)
Neurocognitive disorders	0	0	7 (18%)	4 (13%)
Previous treatments, *n* (%)				
None	19 (48%)	29 (46%)	40 (100%)	31 (100%)
Topical corticosteroids	7 (18%)	11 (17%)	0	0
Oral corticosteroids	7 (18%)	10 (16%)	0	0
Dapsone	5 (13%)	13 (21%)	0	0
Tetracycline	8 (20%)	8 (13%)	0	0
Mycophenolate mofetil	4 (10%)	7 (11%)	0	0

Abbreviations: BP, bullous pemphigoid; BPDAI, bullous pemphigoid disease area index; MMP, mucous membrane pemphigoid; MMPDAI, mucous membrane pemphigoid disease area index; n, number; SD, standard deviation.

* 40 sera analysed both by Olink and Luminex.

** 16 sera analysed both by Olink and Luminex.

### Case series

A total of nine patients, including six men and three women, with severe MMP refractory to conventional therapies, were treated with tocilizumab. The mean age at treatment initiation was 74.8 ± 11.6 years. At the start of tocilizumab therapy, ocular involvement was the most frequently affected site (8/9, 89%), whereas ear‐nose‐throat (ENT) (3/9, 33%) and oral (3/9, 33%) involvement were less common. Direct immunofluorescence (DIF) was positive in 8 of 9 patients (89%), commonly showing linear IgG deposits (6/9, 67%). Serologic tests were negative in all but one case, which showed circulating anti‐BP180 antibodies by enzyme‐linked immunosorbent assay (ELISA). Previous treatments included rituximab (8/9, 89%), dapsone (7/9, 78%), systemic corticosteroids (6/9, 67%) and intravenous immunoglobulin (IVIg) (4/9, 44%). Baseline serum IL‐6 was available in 8/9 patients. During follow‐up, IL‐6 level measurement was performed in 2/9 patients and was measured at month 12 (Patient 1) and month 6 (Patient 2). Five patients had elevated serum levels of IL‐6, while three had IL‐6 levels within the normal range, and one was not measured. The patients' characteristics are displayed in Table 2.

**TABLE 2:** Characteristics of patients treated with tocilizumab.

ID	Sex	Age at start of tocilizumab	Clinical presentation at start of tocilizumab	Prior treatments	DIF results	Serology results	IL‐6 levels (pg/mL), baseline*	Post‐tocilizumab IL‐6 (pg/mL), time point*	Concomitant treatment	Tocilizumab regimen	MMPDAI score (before tocilizumab)	MMPDAI score (last evaluation)	Clinical response	Relapse, time of relapse (month)	Adverse events	Follow‐up (months)
Patient 1	F	85	Ocular	RTX, IV pulse and oral CS, IVIg	IgG, IgA, C3	Negative	4.5	50.9, M12	Oral CS	8 mg/kg IV q4wk	16	3	PR	No	None	24
Patient 2	M	52	Oral, ENT and cutaneous	RTX, oral CS	IgG, IgA, C3	Negative	3.2	22.1, M6	None	8 mg/kg IV q4wk, then q2wk from M12	12	1	PR	No	None	18
Patient 3	M	86	Ocular and ENT	RTX, dapsone	IgG	Negative	Normal	NA	Dapsone	8 mg/kg IV q4wk, reduced to 4 mg/kg at M6	18	7	PR	Yes, 9	Thrombocytopenia (grade II)	11
Patient 4	M	80	Oral, ocular and ENT	RTX, dapsone, IVIg, IV pulse and oral CS	IgA	Negative	229.9	NA	Dapsone	8 mg/kg IV, reduced to 4 mg/kg at M6	21	2.5	PR	No	Hepatic cytolysis (grade I)	12
Patient 5	F	79	Ocular	RTX, dapsone, cyclophosphamide, sulfasalazine	IgG, C3 (lamina lucida, IEM)	Negative	Normal	NA	None	8 mg/kg IV q4wk	10	6	PR	No	Neutropenia (grade I)	6
Patient 6	M	69	Ocular, oral and cutaneous	Dapsone, topical CS	IgG, IgA, C3	Anti‐BP180 Abs, IIF (IgG, epidermal side)	NA	NA	Oral CS	162 mg SC q1wk (concomitant GCA)	10	2	PR	No	None	9
Patient 7	M	85	Ocular	RTX, dapsone, IVIg, IV pulse and oral CS	IgA	Negative	4.7	NA	None	8 mg/kg IV q4wk, reduced to 4 mg/kg at M2	8	6	CDA	No	Neutropenia (grade I)	4
Patient 8	F	74	Ocular	RTX, dapsone, IVIg, oral CS	IgG, IgA, C3	Negative	40	NA	Dapsone	8 mg/kg IV q4wk	28	10	PR	No	None	3
Patient 9	M	63	Ocular	RTX, dapsone, oral CS, doxycycline	Negative	Negative	Normal	NA	Oral CS, IVIg, dapsone, doxycycline	8 mg/kg IV q4wk	10	0.625	CR	No	None	6

*Note*: Normal value for IL‐6: < 2.13 pg/mL. Time points are expressed in months (M) from tocilizumab initiation. MMPDAI scores are reported before tocilizumab initiation and at the last follow‐up evaluation.

Abbreviations: Abs, antibodies; BP180, bullous pemphigoid antigen 180; C3, complement component 3; CDA, control of disease activity; CR, complete remission; CS, corticosteroids; DIF, direct immunofluorescence; ENT, ear, nose and throat; GCA, giant cell arteritis; IEM, immunoelectron microscopy; IgA, immunoglobulin A; IgG, immunoglobulin G; IIF, indirect immunofluorescence; IL‐6, interleukin‐6; IV, intravenous; IVIg, intravenous immunoglobulins; MMPDAI, mucous membrane pemphigoid disease area index; NA, not available; pg/mL, picograms per millilitre; PR, partial remission; q1wk, every week; q2wk, every 2 weeks; q4wk, every 4 weeks; RTX, rituximab; SC, subcutaneous.

* IL‐6 normal value < 2.13 pg/mL.

**FIGURE 2:**
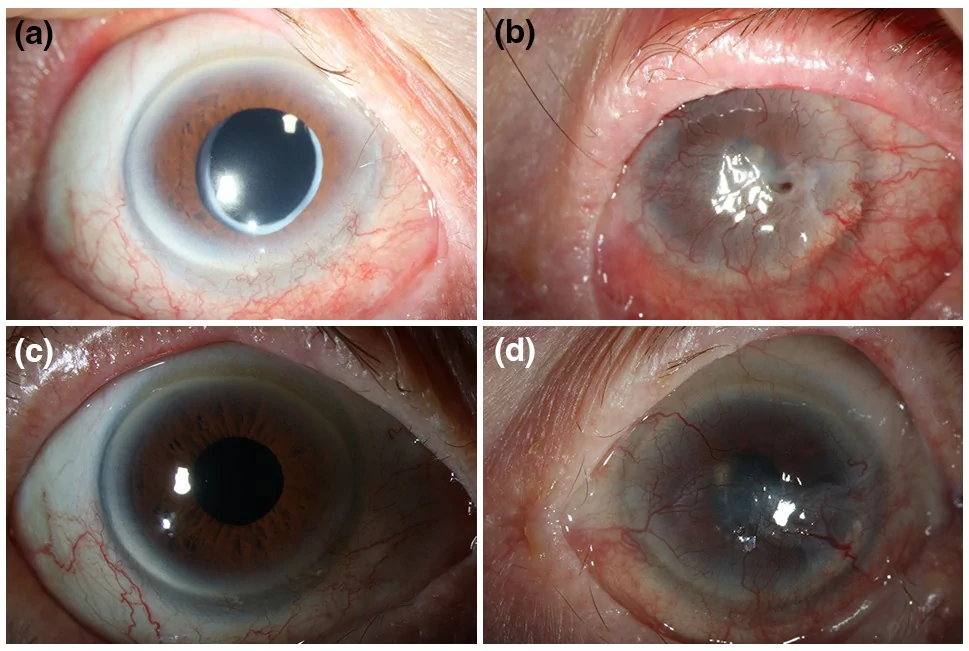
Clinical presentation of patient 1. Changes in ocular involvement in Patient 1 before tocilizumab (a, b) and after 12 months of treatment (c, d), showing a significant reduction of conjunctival inflammation in both eyes and a reduction of corneal opacification in the left eye (d).

**FIGURE 3:**
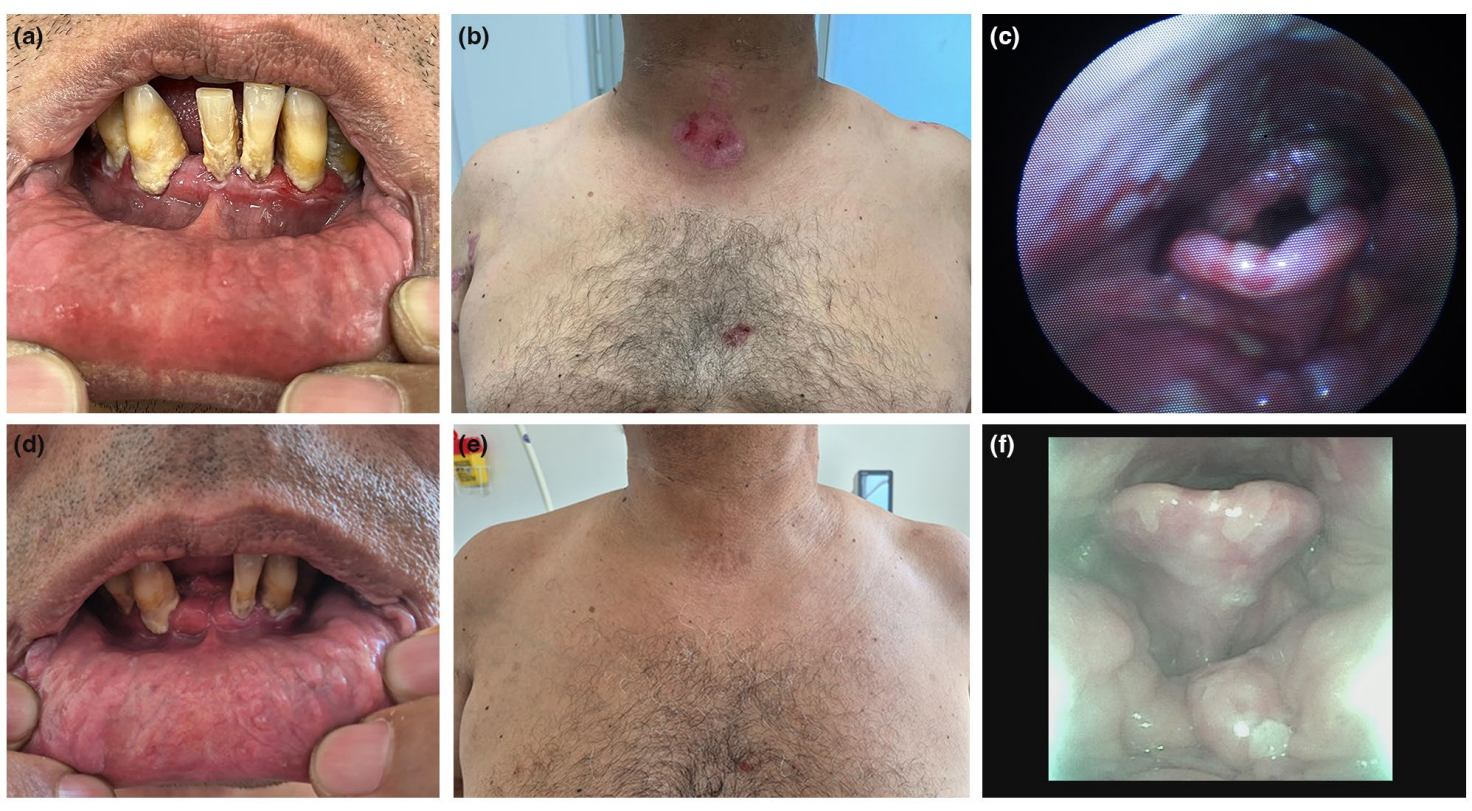
Clinical presentation of patient 2. (a–c) Oral, cutaneous and pharyngolaryngeal involvement prior to tocilizumab treatment. (d–f) After 12 months of treatment, there was complete healing of pharyngolaryngeal and cutaneous erosions.

**FIGURE 4:**
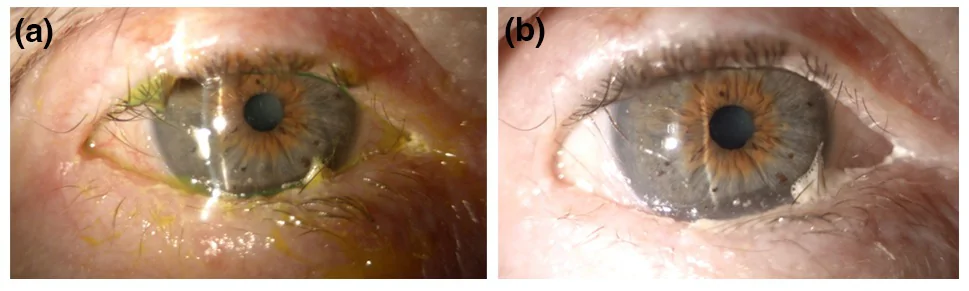
Clinical Presentation of Patient 8. Ocular involvement before tocilizumab (a) and 3 months after initiation of tocilizumab (b).

Tocilizumab was initiated at 8 mg/kg intravenously every 4 weeks in all patients except one, who received subcutaneous tocilizumab due to a concomitant giant cell arteritis. Three patients (33%) were treated exclusively with tocilizumab. Concomitant treatments included oral corticosteroids in three patients (33%) (two started before tocilizumab) and dapsone in four (44%), all started prior to tocilizumab. After initiation of tocilizumab, all patients showed clinical improvement, with a significant reduction in the mean MMPDAI score from 14.8 ± 6.6 to 4.2 ± 3.2 (*p* = 0.009) (Figures 2, 3, 4). At the latest clinical evaluation after a median follow‐up of 9 months after tocilizumab initiation, 7 patients (78%) were in PR, 1 (11%) in CR and 1 (11%) recently treated patient achieved CDA. All 3 patients with normal baseline IL‐6 levels showed clinical improvement, including 2 in PR and 1 in CR. Only one patient had a relapse at month 9, following a dose reduction of tocilizumab related to thrombocytopenia. IL‐6 increased under tocilizumab from 4.5 to 50.9 pg/mL (Patient 1, month 12) and from 3.2 to 22.1 pg/mL (Patient 2, month 6). Four adverse events were reported, all grade I‐II: neutropenia (*n* = 2), thrombocytopenia (*n* = 1) and hepatic cytolysis (*n* = 1). No severe adverse event was observed.

## DISCUSSION

This study suggests that tocilizumab might be considered as a therapeutic option after the failure of conventional treatments in patients with refractory forms of MMP. Our proteomic analysis mainly showed an increase in pro‐inflammatory cytokines (i.e., IL‐6 and IL‐8), which are involved in the activation of T‐cells, and the recruitment and activation of polymorphonuclears, and a decrease in the MMP‐1 metalloproteinase, which has an anti‐fibrotic effect. These findings are in accordance with the pathogenesis of MMP which mainly involves T‐cells and polymorphonuclear neutrophils during the inflammatory phase, and the fibrogenic activity of fibroblasts during the fibrotic phase.^3^, ^6^, ^8^ The rationale for using an anti‐IL‐6 receptor was the observation of increased IL‐6 levels both in Olink and Luminex analyses, whereas IL‐6 is a known driver of fibrosis.^11^ Although serum IL‐6 elevations in our MMP patients were modest, and even within normal levels in 3 of the 9 patients, such results are consistent with observations in other autoimmune diseases showing that clinical response to IL‐6 blockade is not dependent on high baseline IL‐6 concentrations.^12^, ^13^, ^14^

To account for the fact that IL‐6 serum levels can be elevated with ageing, and in patients with neurological comorbidities, we first age‐ and sex‐matched healthy donors with MMP patients.^15^, ^16^ Second, as expected, BP patients were older and had more neurological comorbidities than MMP patients, which could have resulted in higher levels of IL‐6 in patients with BP than in those with MMP. However, we observed the opposite, suggesting that the elevation of IL‐6 in MMP patients relative to those with BP and HD was related to MMP and not to differences in age or frequency of neurological comorbidities. Finally, the levels of the 4 cytokines (i.e., IL‐6, IL‐8, MIP‐1α/CCL3 and MIP‐1β/CCL4) decreased in patients who achieved a CR as compared with those who had persistent disease activity, suggesting a relationship between changes in these cytokine levels and the clinical course of MMP.

Importantly, these findings distinguish MMP from BP at the proteomic level. MMP was characterized by elevated pro‐inflammatory chemokines (IL‐8, MIP‐1α/CCL3, MIP‐1β/CCL4) and IL‐6, consistent with innate immune activation and neutrophil recruitment, in line with the enrichment of chemokine and NOD‐like receptor signalling pathways observed in MMP. In contrast, BP was characterized by higher expression of soluble CD30 (AUC = 0.27, adjusted *p* = 0.01, Luminex) and MCP‐4/CCL13 (AUC = 0.209, adjusted *p* < 0.001, Olink), two Th2‐associated markers, consistent with the eosinophil‐rich inflammatory infiltrate characteristic of BP.^17^, ^18^

We used tocilizumab because it has a good safety profile, and no drugs inhibiting the other cytokines found to be elevated were currently available. Due to the very small size of laryngeal and conjunctival biopsies, direct in situ quantification of IL‐6 could not be performed. However, the significant clinical efficacy of tocilizumab strongly suggested a local IL‐6–driven effect. Indeed, it has been previously demonstrated that IL‐6 is significantly upregulated in conjunctival tissues of patients with ocular MMP.^19^ Our findings and several reports from the literature further support the hypothesis that IL‐6–mediated inflammation contributes to persistent ocular surface damage and fibrosis, independently of autoantibody status, which may explain the benefit of IL‐6 blockade in seronegative cases.^20^, ^21^

Indeed, tocilizumab resulted in a considerable clinical improvement of various mucosal sites including the eyes and pharyngolaryngeal erosions in these nine patients, while ocular disease is the most devastating and notoriously difficult‐to‐treat mucosal site of MMP, and laryngeal lesions may be life‐threatening and are responsible for a higher mortality rate of MMP patients relative to the general population.^22^ In all patients, tocilizumab was used as a second‐ or third‐line treatment, raising the question of the potential effect of previous treatments, in particular rituximab and IVIg. However, the efficacy of tocilizumab was strongly suggested by the fact that the patients' condition rapidly improved under tocilizumab, while ocular and ENT lesions in particular had worsened with the previously used treatments (in particular, rituximab, oral corticosteroids and dapsone). Additionally, tocilizumab was used alone in three patients and allowed a rapid tapering of corticosteroid doses in those receiving concomitant oral corticosteroids. Additionally, clinical improvement was evidenced by a significant decrease in the MMPDAI score. Post‐treatment serum IL‐6 measurement was available in only 2/9 patients; both showed an increase in circulating IL‐6 under tocilizumab, consistent with the well‐described pharmacological effect of IL‐6 receptor blockade leading to an increase in free serum IL‐6.^12^

The mechanism of action involves IL‐6 binding to its receptor, which activates JAK kinases, leading to the phosphorylation of STAT3 and STAT1.^11^, ^23^, ^24^ Interestingly, JAK inhibitors have been successfully tested in several cases of refractory ocular MMP.^25^, ^26^, ^27^ However, tocilizumab provides an advantage due to its favourable safety profile, especially since it does not share the cardiovascular and thrombotic events of JAK inhibitors, which is of particular interest in elderly patients and those with cardiovascular risk factors.^28^, ^29^

One of the limitations of the proteomic analyses might be the inclusion of previously treated patients with MMP, although the serum level of the four key cytokines did not differ at baseline between treatment‐naïve and previously treated patients. Another limitation of this translational study is the limited number of patients treated with tocilizumab as a second‐ or third‐line therapy, which is related to the rarity of MMP and even more so of severe forms, as reported in these nine patients. Consequently, the efficacy and safety of tocilizumab in MMP remain to be confirmed in larger, prospective studies.

In conclusion, our findings support the further evaluation of IL‐6 blockade in cases of severe and refractory MMP. Tocilizumab could be considered on a case‐by‐case basis following the failure of established therapies (e.g., rituximab, IVIg and cyclophosphamide).

## AUTHOR CONTRIBUTIONS

Conceptualization: B.T., M.M.‐V., P.J. Methodology: B.T., M.M.‐V., A.G., P.J. Formal analysis: B.T., A.G. Investigation: B.T., M.M.‐V., J.G., M.‐L.G., M.M., B.B., G.C., P.B., A.T., H.M., C.M., A.V., E.‐P.L., C.L.R.‐V., M.C., S.C., P.J. Data curation: B.T., A.G. Writing – original draft: B.T., P.J. Writing – review & editing: M.M.‐V., J.G., M.‐L.G., M.M., B.B., G.C., P.B., S.B., A.T., H.M., C.M., A.V., E.‐P.L., C.L.R.‐V., M.C., O.B., V.H., S.C., A.G. Supervision: P.J. Funding acquisition: M.M.‐V., P.J., B.T.

## FUNDING INFORMATION

Funded by the French Society of Dermatology and FIMARAD.

## CONFLICT OF INTEREST STATEMENT

P. Joly reports consulting fees and/or advisory board participation from Amgen, Argenx, AstraZeneca, Thermo Fisher, Sanofi, Akari, Janssen, Novartis, Servier, Chugai, Kezar Life Science, Regeneron, UCB, Principia Biopharma, Zenyaku Kogyo, Dualyx, JJP Biologics and Lilly, and travel support from Janssen. C. Le Roux‐Villet reports honoraria from Janssen and Amgen, advisory board participation for Almirall and travel support from UCB, Lilly, Almirall and LEO Pharma. O. Boyer reports grants from Argenx, consulting fees from Argenx and Egle Tx, honoraria from BMS and CSL Behring, and advisory board participation for Premier Research. All other authors declare no conflicts of interest.

## ETHICAL APPROVAL

The study protocol was reviewed and approved by the local ethics committee. Patients' sera were obtained from the approved trial RITUX‐MMP (ClinicalTrials.gov number NCT03295383) and local autoimmune blistering disease collection (DC‐2021‐4499).

## ETHICS STATEMENT

This study was conducted in accordance with the ethical standards of the relevant institutional ethics committee. Written informed consent for the publication of clinical photographs was obtained from all patients.

## Supporting information


**Table S1**. Comparison of proteomic expression between patients with MMP and HD.
**Table S2**. Comparison of proteomic expression between patients with MMP and patients with BP.
**Table S3**. Proteomic pathway analysis of patients with MMP compared to HD and patients with BP.
**Table S4**. Differential luminex assay results in patients with MMP versus HD.
**Table S5**. Differential luminex assay results in patients with MMP versus BP.

**Figure S1.** Volcano Plots of Differential Protein Expressions. Volcano Plots representing significant differential proteins between MMP versus HD (A) and MMP versus BP (B). Difference (X‐axis) is expressed by the Hodges‐Lehman estimator (associated with Mann–Whitney tests) on the Olink protein expression scale without z‐transformation. The y‐axis represents unadjusted *p*‐values. Abbreviations: HD, Healthy Donors; BP, Bullous Pemphigoid; MMP, Mucous Membrane Pemphigoid.
**Figure S2**. Comparison of Protein Concentrations Measured by Luminex between patients with MMP and HD. The medians (with interquartile ranges [IQR]) of significantly decreased (A) and increased (B) proteins in patients with MMP (*n* = 63) compared to HD (*n* = 24) are shown. Abbreviations: HD, Healthy Donors; MMP, Mucous Membrane Pemphigoid.
**Figure S3**. Comparison of Protein Concentrations Measured by Luminex between patients with MMP and BP. The medians (with interquartile ranges [IQR]) of significantly decreased (A) and increased (B) proteins in patients with MMP (n = 63) compared to patients with BP (*n* = 31) are shown. Abbreviations: BP, Bullous Pemphigoid; MMP, Mucous Membrane Pemphigoid.
**Figure S4**. Comparison of IL‐6, IL‐8, MIP‐1α/CCL3 and MIP‐1β/CCL4 Concentrations Measured by Luminex between Treatment‐Naïve and Previously Treated Patients with MMP. At baseline, serum IL‐6, IL‐8, MIP‐1α/CCL3 and MIP‐1β/CCL4 did not differ between treatment‐naïve (*n* = 29) and previously treated patients with MMP (*n* = 34). Abbreviations: MMP, Mucous Membrane Pemphigoid.
**Figure S5**. Comparison of IL‐6, IL‐8, MIP‐1α/CCL3 and MIP‐1β/CCL4 Concentrations Measured by Luminex between Patients with MMP in CR and AD. Patients with MMP who achieved CR after treatment (*n* = 24) had significantly lower serum IL‐6 (*p* < 0.0001), IL‐8 (*p* = 0.0382), MIP‐1α/CCL3 (*p* = 0.0005) and MIP‐1β/CCL4 (*p* = 0.0009) than patients with persistent AD (*n* = 20). Abbreviations: AD, Active Disease; CR, Complete Remission; MMP, Mucous Membrane Pemphigoid.

## Data Availability

The data that support the findings of this study are available from the corresponding author upon reasonable request.
