# Supplementary material for: Tocilizumab for severe and refractory mucous membrane pemphigoid
Source: J Eur Acad Dermatol Venereol. 2026 May 27;40(10):1682–91. doi: 10.1111/jdv.70510 (PMC13613347; doi:10.1111/jdv.70510)
Supplement: Supplementary file 1 [file JDV-40-1682-s001.docx]

**Supplementary Online Content**

**eFigure 1. Volcano Plots of Differential Protein Expressions.** Volcano Plots representing significant differential proteins between MMP vs HD (A) and MMP vs BP (B). Difference (X-axis) is expressed by Hodges-Lehman estimator (associated to Mann-Whitney tests) on the Olink protein expression scale without z-transformation. Y-axis represents unadjusted P-values. *Abbreviations: MMP, Mucous Membrane Pemphigoid; HD, Healthy Donors; BP, Bullous Pemphigoid.*

**eFigure 2. Comparison of Protein Concentrations Measured by Luminex between patients with MMP and HD.** The medians (with interquartile ranges [IQR]) of significantly decreased (A) and increased (B) proteins in patients with MMP (n=63) compared to HD (n=24) are shown. *Abbreviations: MMP, Mucous Membrane Pemphigoid; HD, Healthy Donors.*

**eFigure 3. Comparison of Protein Concentrations Measured by Luminex between patients with MMP and BP.** The medians (with interquartile ranges [IQR]) of significantly decreased (A) and increased (B) proteins in patients with MMP (n=63) compared to patients with BP (n=31) are shown. *Abbreviations: MMP, Mucous Membrane Pemphigoid; BP, Bullous Pemphigoid.*

**eFigure 4. Comparison of IL-6, IL-8, MIP-1α/CCL3, and MIP-1β/CCL4 Concentrations Measured by Luminex between Treatment-Naïve and Previously Treated Patients with MMP.** At baseline, serum IL-6, IL-8, MIP-1α/CCL3, and MIP-1β/CCL4 did not differ between treatment-naïve (n=29) and previously treated patients with MMP (n=34). *Abbreviations: MMP, Mucous Membrane Pemphigoid.*

**eFigure 5. Comparison of IL-6, IL-8, MIP-1α/CCL3, and MIP-1β/CCL4 Concentrations Measured by Luminex between Patients with MMP in CR and AD.** Patients with MMP who achieved CR after treatment (n=24) had significantly lower serum IL-6 (P<0.0001), IL-8 (P=0.0382), MIP-1α/CCL3 (P=0.0005), and MIP-1β/CCL4 (P=0.0009) than patients with persistent AD (n=20). *Abbreviations: AD, Active Disease; CR, Complete Remission; MMP, Mucous Membrane Pemphigoid.*

**eFigure 1. Volcano Plots of Differential Protein Expressions.** Volcano Plots representing significant differential proteins between MMP vs HD (A) and MMP vs BP (B). Difference (X-axis) is expressed by Hodges-Lehman estimator (associated to Mann-Whitney tests) on the Olink protein expression scale without z-transformation. Y-axis represents unadjusted P-values. *Abbreviations: MMP, Mucous Membrane Pemphigoid; HD, Healthy Donors; BP, Bullous Pemphigoid.*

**A**

**B**

**eFigure 2. Comparison of Protein Concentrations Measured by Luminex between patients with MMP and HD.** The medians (with interquartile ranges [IQR]) of significantly decreased (A) and increased (B) proteins in patients with MMP (n=63) compared to HD (n=24) are shown. *Abbreviations: MMP, Mucous Membrane Pemphigoid; HD, Healthy Donors.*

**eFigure 3. Comparison of Protein Concentrations Measured by Luminex between patients with MMP and BP.** The medians (with interquartile ranges [IQR]) of significantly decreased (A) and increased (B) proteins in patients with MMP (n=63) compared to patients with BP (n=31) are shown. *Abbreviations: MMP, Mucous Membrane Pemphigoid; BP, Bullous Pemphigoid.*

**eFigure 4. Comparison of IL-6, IL-8, MIP-1****α/CCL3, and MIP-1****β/CCL4 Concentrations Measured by Luminex between Treatment-Naïve and Previously Treated Patients with MMP.** At baseline, serum IL-6, IL-8, MIP-1α/CCL3, and MIP-1β/CCL4 did not differ between treatment-naïve (n=29) and previously treated patients with MMP (n=34). *Abbreviations: MMP, Mucous Membrane Pemphigoid.*

**eFigure 5. Comparison of IL-6, IL-8, MIP-1α/CCL3, and MIP-1β/CCL4 Concentrations Measured by Luminex between Patients with MMP in CR and AD.** Patients with MMP who achieved CR after treatment (n=24) had significantly lower serum IL-6 (P<0.0001), IL-8 (P=0.0382), MIP-1α/CCL3 (P=0.0005), and MIP-1β/CCL4 (P=0.0009) than patients with persistent AD (n=20). *Abbreviations: AD, Active Disease; CR, Complete Remission; MMP, Mucous Membrane Pemphigoid.*
